# Sequence variation does not confound the measurement of plasma PfHRP2 concentration in African children presenting with severe malaria

**DOI:** 10.1186/1475-2875-11-276

**Published:** 2012-08-16

**Authors:** Thiranut Ramutton, Ilse CE Hendriksen, Juliet Mwanga-Amumpaire, George Mtove, Rasaq Olaosebikan, Antoinette K Tshefu, Marie A Onyamboko, Corine Karema, Kathryn Maitland, Ermelinda Gomes, Samwel Gesase, Hugh Reyburn, Kamolrat Silamut, Kesinee Chotivanich, Kamoltip Promnares, Caterina I Fanello, Lorenz von Seidlein, Nicholas PJ Day, Nicholas J White, Arjen M Dondorp, Mallika Imwong, Charles J Woodrow

**Affiliations:** 1 Department of Clinical Tropical Medicine, Faculty of Tropical Medicine, Mahidol University, Bangkok, Thailand; 2 Mahidol-Oxford Tropical Medicine Research Unit, Faculty of Tropical Medicine, Mahidol University, Bangkok, Thailand; 3 Centre for Clinical Vaccinology and Tropical Medicine, Churchill Hospital, University of Oxford, Oxford, UK; 4 Mbarara University of Science and Technology and Epicentre Research Base, Mbarara, Uganda; 5 National Institute for Medical Research, Amani Centre, Tanga, Tanzania; 6 MRC laboratories, Banjul, The Gambia; 7 Kinshasa School of Public Health, Kingasani Research Centre, Kinshasa, DRC; 8 Malaria Control Program, Ministry of Health, Kigali, Rwanda; 9 Kenya Medical Research Institute (KEMRI)–Wellcome Trust Research Programme, Kilifi, Kenya; 10 Hospital Central da Beira, Beira, Mozambique; 11 National Institute for Medical Research, Korogwe Research Laboratory, Tanga, Tanzania; 12 London School of Tropical Medicine and Hygiene, London, UK; 13 Department of Molecular Biotechnology and Bioinformatics, Faculty of Science, Prince of Songkla University, Hat Yai, Songkhla, 90112, Thailand; 14 Center for Genomics and Bioinformatics Research, Faculty of Science, Prince of Songkla University, Hat Yai, Songkhla, 90112, Thailand; 15 Menzies School of Health Research, Casuarina, NT, Australia; 16 Department of Molecular Tropical Medicine and Genetics, Faculty of Tropical Medicine, Mahidol University, Bangkok, Thailand

**Keywords:** Malaria, Falciparum, Severe, Africa, Histidine-rich protein, Tandem repeat

## Abstract

**Background:**

*Plasmodium falciparum* histidine-rich protein PFHRP2 measurement is used widely for diagnosis, and more recently for severity assessment in falciparum malaria. The *Pfhrp2* gene is highly polymorphic, with deletion of the entire gene reported in both laboratory and field isolates. These issues potentially confound the interpretation of PFHRP2 measurements.

**Methods:**

Studies designed to detect deletion of *Pfhrp2* and its paralog *Pfhrp3* were undertaken with samples from patients in seven countries contributing to the largest hospital-based severe malaria trial (AQUAMAT). The quantitative relationship between sequence polymorphism and PFHRP2 plasma concentration was examined in samples from selected sites in Mozambique and Tanzania.

**Results:**

There was no evidence for deletion of either *Pfhrp2* or *Pfhrp3* in the 77 samples with lowest PFHRP2 plasma concentrations across the seven countries. *Pfhrp2* sequence diversity was very high with no haplotypes shared among 66 samples sequenced. There was no correlation between *Pfhrp2* sequence length or repeat type and PFHRP2 plasma concentration.

**Conclusions:**

These findings indicate that sequence polymorphism is not a significant cause of variation in PFHRP2 concentration in plasma samples from African children. This justifies the further development of plasma PFHRP2 concentration as a method for assessing African children who may have severe falciparum malaria. The data also add to the existing evidence base supporting the use of rapid diagnostic tests based on PFHRP2 detection.

## Background

The *Plasmodium falciparum* histidine-rich proteins PFHRP2 and PFHRP3 [1] are soluble proteins containing highly antigenic tandem repeat sequences [2] and are produced in large quantities during the asexual blood stage of the *P. falciparum* lifecycle [3] where their concentration represents a dynamic equilibrium [4]. Orthologs are not found in other species of human malaria [5]. Antibody detection of PFHRP2 (which also picks up PFHRP3) is, therefore, a sensitive method for the rapid diagnosis of *P. falciparum* malaria using whole blood [6], as well as an *in vitro* drug sensitivity readout that has found increasing use in recent years [7]. PFHRP2 is also released into plasma, where its concentration reflects the extent of current and previous cycle parasite sequestration, a process considered critical to the pathology of severe malaria [8].

Since the initial description of these histidine-rich proteins more than two decades ago [1,9], their physiological role in the lifecycle of *P. falciparum* has been subject to a number of lines of investigation, with the focus of most attention on haem polymerization [10]. A degree of redundancy exists with parasites lacking one or both of these proteins still able to propagate in asexual stage culture and complete the entire parasite life cycle [11-13]. Nevertheless, classical falciparum genetic crosses in which one parent had a deletion of *Pfhrp3* demonstrated complete [14] or partial [15] bias towards *Pfhrp3* inheritance (although the cross-over interval was not fine enough to attribute enhanced fitness specifically to *Pfhrp3* as opposed to nearby genes). Perhaps surprisingly, *Pfhrp2* did not show inheritance bias in the HB3 x Dd2 cross [15]; low-level transcription in the HB3 parent [16] could potentially explain this in part or in whole.

In contrast to these studies on cultured isolates, prior to 2010 no field isolates had been reported which lacked either *Pfhrp2* or *Pfhrp3*. However, relatively recent evidence of significant numbers of parasites with deletion of one or both genes has emerged in the Peruvian Amazon [17,18], generating concerns over the reliability of PFHRP2-based rapid diagnostic tests (RDTs) in this region. There has been little evidence that deletion of either gene occurs to a significant degree in Africa. This may reflect both the true rarity of the event (given the predicted deleterious effect on fitness), along with the higher multiplicities of infection found in high transmission settings. It is therefore concerning that isolates from Mali lacking *Pfhrp2* have now been described [19].

Both *Pfhrp2* and *Pfhrp3* also exhibit extensive insertion-deletion polymorphism, and the potential for this to reduce the accuracy of RDTs has been examined at national [20] and global scales [21,22]. However, whether sequence variation is an important confounder in the assessment of plasma concentration, and hence disease severity, has not been studied. This study examined the relationship between *Pfhrp2* sequence and plasma PFHRP2 concentration in samples derived from a large group of African children treated for severe malaria in the AQUAMAT trial [23].

## Methods

A PCR-based approach was used to answer a number of related questions. First, *Pfhrp2* deletions were sought in parasites from patients admitted with a diagnosis of severe malaria but who had very low or undetectable plasma PFHRP2 levels by ELISA. Deletions in *Pfhrp3* were also checked since by cross-reacting with PFHRP2, the less abundant PFHRP3 allows RDTs to retain some sensitivity even when *Pfhrp2* deletions are present. Finally, the relationship between *Pfhrp2* exon 2 length and repeat structure and plasma concentration was explored.

### Samples

Samples were obtained from children enrolled in the AQUAMAT trial, a large multinational trial comparing quinine and artesunate for the treatment of severe malaria in African children undertaken between October 2005 and July 2010 and described in detail elsewhere [23]. Children were included if they showed signs of severe malaria (defined by clinical criteria) and had a positive *P. falciparum* lactate dehydrogenase RDT. Patients were excluded if treated parenterally for more than 24 hours before admission. Patients were randomized to treatment with either parenteral artesunate or quinine. Baseline blood samples included a peripheral blood slide. Slide reading was performed by microscopists at the Mahidol-Oxford Tropical Medicine Research Unit and parasitaemia/μl was calculated from thin film (count/1,000 RBCx125.6xHct) or thick film (count/200 WBCx40). Dried whole blood spots were used for molecular studies. EDTA blood for plasma PFHRP2 measurement was received from nine of the 11 AQUAMAT research sites in seven countries (Table 1). 

**Table 1:** **Selection of samples examined for possible *Pfhrp2 *and *Pfhrp3 *deletion**

**Country**	**Site**	**Available samples**	**Samples tested (%)**
DRC	Kinshasa	420	6 (1.43)
The Gambia	Banjul	60	2 (3.33)
Kenya	Kilifi	378	12 (3.17)
Mozambique	Beira	659	19 (2.88)
Rwanda	Kigali	170	10 (5.88)
	Nyanza	111	5 (4.50)
Tanzania	Korogwe	534	3 (0.56)
	Teule	901	15 (1.66)
Uganda	Mbarare	593	5 (0.84)
Total		3826	77 (1.98)

DNA samples from established *P. falciparum* lines (K1, Dd2, 3d7) were provided by MR4 and used to control *Pfhrp2* and *Pfhrp3* PCR results. A number of samples from within Asia were also tested to validate methodology, confirming the finding [22] that *Pfhrp2* exon 2 sequences from African samples are longer than those from Cambodia, as they have substantially more type 7 repeats. To reduce the potential confounding effect of differing clinical characteristics in patients recruited in different sites, the relationship between *Pfhrp2* sequence and plasma concentration was focused on sample sets from Mozambique and Tanzania (Teule).

Appropriate ethical approvals were granted by the Oxford Tropical Research Ethics Committee, the Ethics Committee of the Faculty of Tropical Medicine, Mahidol University and the individual study site countries’ ethical review boards.

### Laboratory techniques

Parasite DNA from dried blood spots was extracted via the QIAmp DNA Mini kit using the standard protocol and stored at −20°C until use. For both *Pfhrp2* and *Pfhrp3*, a semi-nested PCR approach was used involving identical primers and conditions to those already described by Baker *et al.*[21]. These primers anneal to the conserved end regions of exon 2 of *Pfhrp2* and *Pfhrp3*, with the intervening sequence encompassing the variable histidine-rich repeats of these genes. To confirm *P. falciparum* infection in *Pfhrp2*-negative samples or those with very weak bands, a fully nested PCR *Plasmodium* species detection approach was employed using specific oligonucleotide primers to amplify a segment of ssrRNA using published primers and conditions [24]. For *Pfhrp2* sequencing, amplified DNA products were purified using the Favorgen Gel/PCR purification kit according to the manufacturer’s instructions. DNA concentrations were estimated by agarose gel electrophoresis and samples sent for sequencing (using the *Pfhrp2* internal forward primer [21]) at Macrogen, Seoul, South Korea.

Plasma PFHRP2 was measured in freeze-thawed EDTA plasma samples according to standard techniques [25]. In brief a commercial sandwich ELISA kit (Celisa, Cellabs, Sydney, Australia) was used according to the manufacturer’s instructions with minor modifications and blinded to patient outcomes [8]. Pooled reference plasma was derived from 20 subjects with parasitaemia >200,000/μl and used to construct standard curves.

### Statistics

Comparisons of *Pfhrp2* exon 2 length and repeat number were undertaken by the Mann–Whitney test in GraphPad Prism.

## Results

### Assessment of possible deletion of *Pfhrp2* or *Pfhrp3*

**Figure 1:**
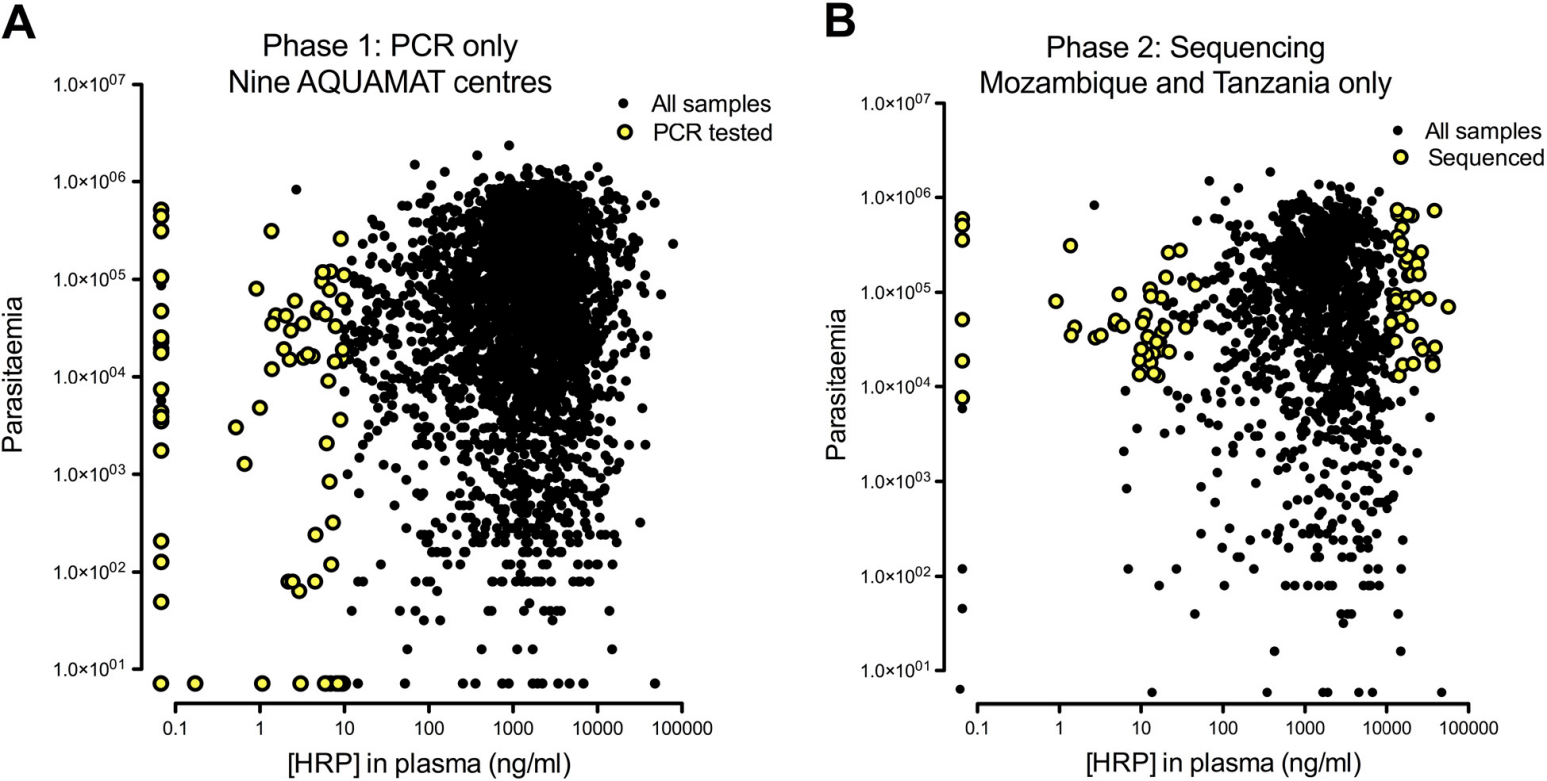
** PfHRP2 levels and parasitaemias for samples examined. A**: samples subjected to PCR for *Pfhrp2* and *Pfhrp3* across all relevant AQUAMAT centres; **B**: samples sequenced from Mozambique and Tanzania (Teule site)

Plasma PFHRP2 was measured in 3,826 of the 5,426 children with pLDH-RDT-confirmed falciparum malaria included in the AQUAMAT trial. The 80 samples associated with lowest plasma PFHRP2 levels yielded 77 blood spots which were analysed using a semi-quantitative approach for possible deletions in *Pfhrp2* or *Pfhrp3* (Table 1, Figure 1a). These samples had a median parasitaemia of 9,043/μl (compared to 79,397/μl for the whole dataset). PCR for *Pfhrp2* revealed bands of the anticipated size in 70 cases; in seven cases (median parasitaemia 3,040/μl) no band could be obtained despite repeated attempts. In all these *Pfhrp2*-negative cases, the sample also had negative or very weak bands for *Pfhrp3* as well as species identification, indicating that DNA content of the sample was at or below the threshold of detection. In an analogous manner, PCR for *Pfhrp3* was found to be positive in 67 samples, and was only negative (ten cases) when the other two reactions were negative or produced very dilute products.

### Sequence polymorphism in *Pfhrp2*

In order to explore the relationship between *Pfhrp2* sequence and plasma concentration, the aim was to obtain 80 sequences from patients in Tanzania (Teule) and Mozambique associated with extreme values of plasma PFHRP2 (40 high, 40 low) but with relatively high peripheral parasitaemias (greater than 10,000/μl) and hence likely to produce reliable material for sequencing (Figure 1b). Twenty-six clean PCR products suitable for sequencing had already been obtained from these two sites in the first phase of testing samples with low PFHRP2 levels. An additional 62 (22 low PFHRP2, 40 high) samples therefore underwent DNA extraction and *Pfhrp2* PCR; all gave bands with no evidence of *Pfhrp2* deletion. After sequencing, clear and complete exon 2 sequences were available in 34 samples from Mozambique and 32 from Tanzania. Across these 66 samples, 37 patients had low PFHRP2 levels (median (range) = 10.9 ng/ml (undetectable – 46.1)) while 29 had high PFHRP2 levels (18,017 ng/ml (11,289–56,818)).

**Figure 2:**
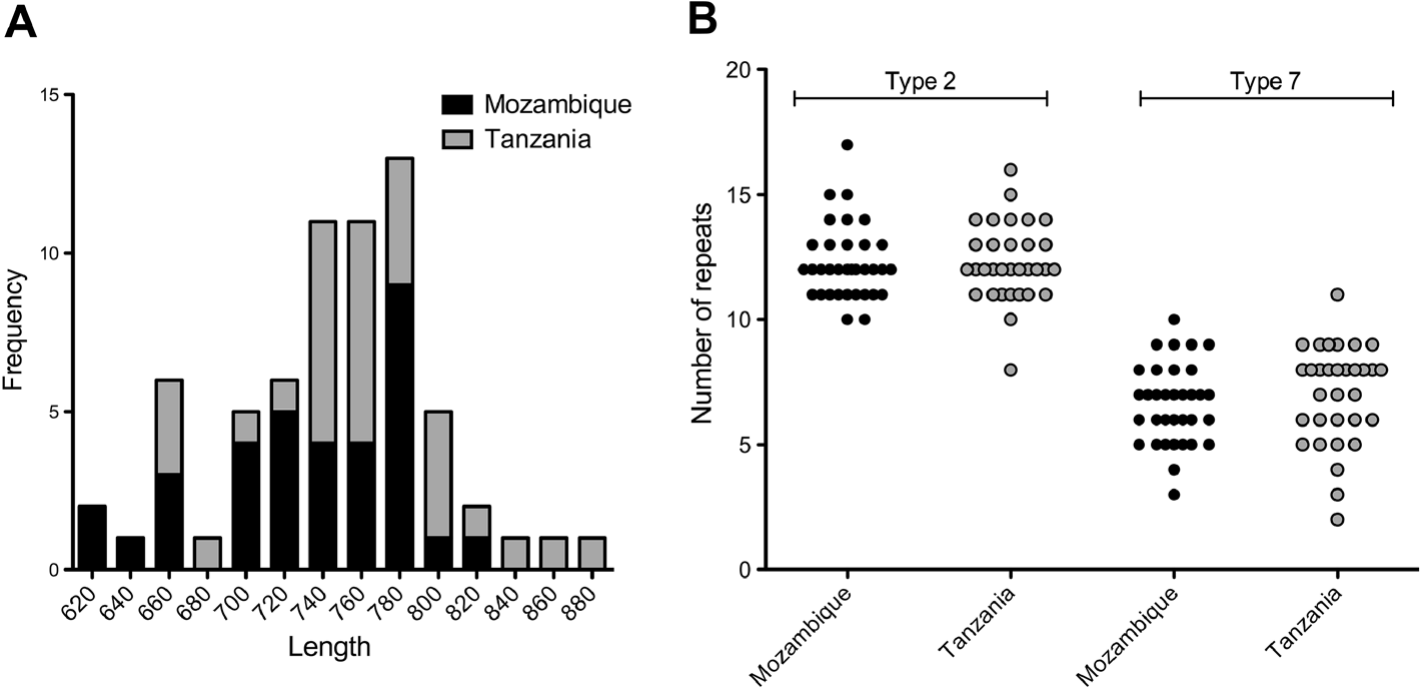
*** Pfhrp2 *exon 2 length and repeat numbers according to country.A**: frequency of each exon size (bin width of 20 bp); **B**: numbers of type 2 and 7 repeats in each sample

As expected from the gel electrophoresis results, and consistent with previous findings [20-22], PCR products were highly variable in length, as well as the numbers and types of repeat (Figure 2a). For Mozambique the median *Pfhrp2* exon 2 PCR product length was 741 bp (range 627–816) and for Tanzania 759 (range 651–876) (p = 0.12; Table 2). Of the repeat types described by Baker *et al.*, types 2 (AHHAHHAAD) and 7 (AHHAAD) were the most prominent (Figure 2b), constituting more than half of each sequence; these were also the only repeat types observed in all samples. All the other repeats documented were fewer in number (no more than six of any repeat in any sample). There was no significant difference in the number of type 2, type 7 or any other repeat between Mozambique and Tanzania (Figure 2b, Table 2) and the two datasets were pooled to explore the possible association between *Pfhrp2* polymorphism and plasma levels. 

**Table 2:** ***Pfhrp2* exon 2 length and repeat polymorphism data by country**

Population	Mozambique	Tanzania (Teule)	Combined	P value
Total number	34	32	66	
Length: median (range)	741 (627–816)	759 (651–876)	751.5 (627–876)	0.12
Haplotypes	34	32	66	
Repeats				
Type 2	12 (10–17)	12 (8–16)	12 (8–17)	0.70
Type 7	7 (3–10)	8 (2–11)	7 (2–11)	0.22

**Figure 3:**
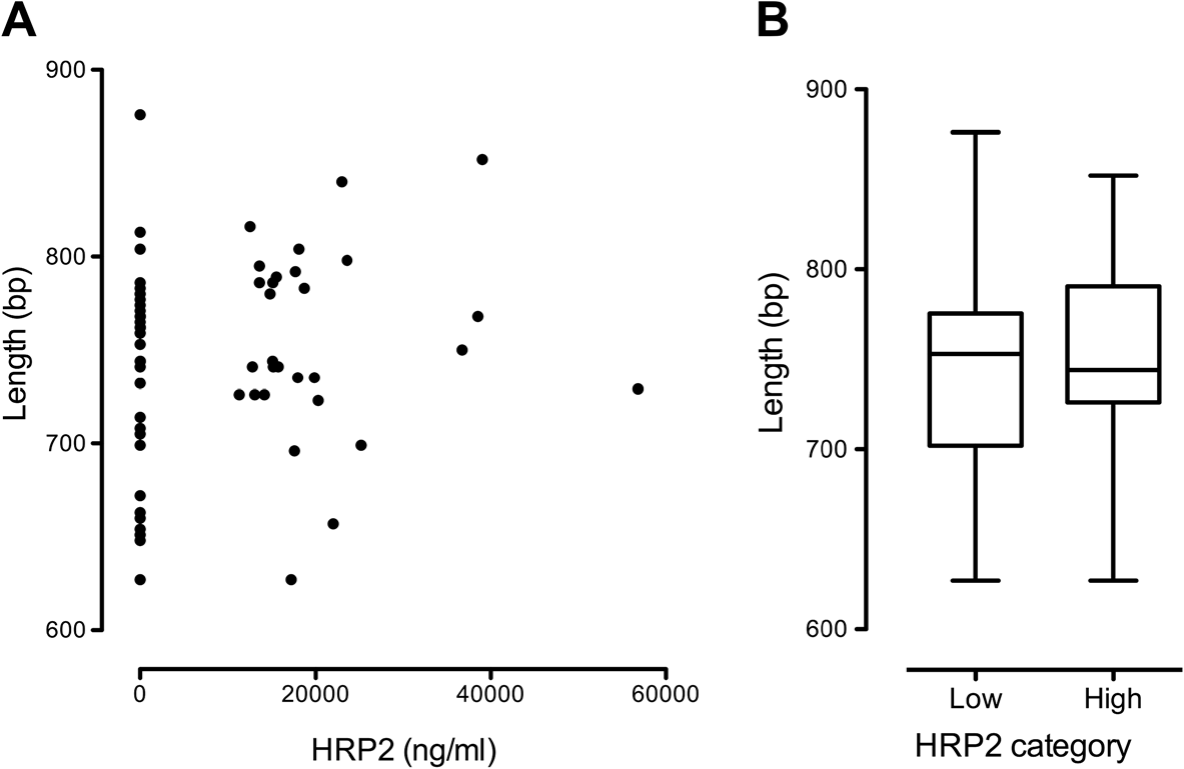
** Relationship between *Pfhrp2 *exon 2 length and plasma level.A**: individual samples; **B**: categorized by PFHRP2 level

Sequence length, number of type 2 repeats and number of type 7 repeats did not correlate with PFHRP2 plasma concentration when expressed as a continuous variable (Figure 3a). There was also no significant difference in these sequence properties between low and high categories of PFHRP2 (Figure 3b).

## Discussion

PFHRP2 is released into the circulating plasma [8,26-28] where it persists for several days [29]. Because of the exponential rise in parasitaemia that occurs during the development of *P. falciparum* in the blood, in whole blood a large portion of PFHRP2 is located within asexual parasites [27], and whole-blood PFHRP2 concentrations correlate, therefore, with peripheral blood parasitaemia. By contrast, plasma PFHRP2 provides a summative picture of previous cycles of growth that reflects sequestered parasite biomass rather than circulating biomass [8]. Plasma PFHRP2 measurement can therefore potentially be applied in the management of severely ill patients in high transmission settings who may be ill because of malaria or alternatively have another severe illness, with malaria parasitaemia an incidental finding [30]. Post-mortem reveals that 25% of patients thought to have died of severe malaria have other serious pathology [31]. Confident differentiation of severe malaria from other pathologies where the prevalence of malaria parasitaemia is high is very difficult [30]; observation of malarial retinopathy improves the accuracy of diagnosis for cerebral malaria [32], but requires skill and training and is rarely achievable in practice outside centres of excellence. Since plasma PFHRP2 derives from sequestered parasites that are critical to the pathology of severe malaria, it has potential to differentiate ‘true’ severe malaria from alternative illnesses in parasitaemic patients, as well as provide an independent marker of prognostic significance in severe malaria [8]. Plasma PFHRP2 levels were found to be higher in patients with severe falciparum malaria compared to uncomplicated malaria in Indonesia [33] and Thailand [8] although studies in Kenya [34] and Papua New Guinea [35] did not find a significant difference. Explanations for these apparently discrepant findings include different classifications of disease severity as well as differences in the intensity of transmission. A particular problem in these studies, and in other studies of severe malaria is the classification of children with severe anaemia and low parasitaemia as having “severe malaria”, many of whom have chronic anaemia and incidental parasitaemia. These patients do not have large numbers of sequestered parasites and so do not have high plasma PFHRP2 concentrations.

The antibodies present in PFHRP2 RDT kits detect the repetitive histidine-rich repeats encoded by exon 2 of *Pfhrp2* (and *Pfhrp3*) [2,36]. This is a strength of PFHRP2-based diagnosis, since the antigen sequence appears many times within the protein and one protein can be bound by several antibody molecules potentially leading to amplification of signal and high assay sensitivity. However, the nature of the antigen detected optimally by each RDT clearly differs between kits [2,36]. The possibility that this might impair the accuracy of RDTs was reflected for a time by a concern that certain patterns of repeat polymorphism (relating to numbers of type 2 and type 7 repeats) were associated with lower levels of detection, based on a semi-quantitative analysis of kit sensitivity [21]. Subsequent work has not supported this possibility and the performance of a range of RDTs now appears unlikely to be confounded by sequence polymorphisms [22].

This study aimed to address the concern that quantification of plasma PFHRP2 might be confounded by sequence polymorphism in terms of type and number of histidine-containing repeats in PFHRP2. Among parasites from Mozambique and Tanzania, a diverse pattern of repeat types was observed consistent with previous studies from a wide range of African countries [20-22]. Within this variation, there was no relationship between the number of repeats observed, or overall sequence length, and plasma PFHRP2 level, mirroring the semi-quantitative findings from studies examining rapid diagnostic tests [22]. These findings do not support the idea that sequence polymorphism has a substantial influence on ELISA-based PFHRP2 quantitation in African samples. The results presented here, along with existing data that in Southeast Asian adult plasma PFHRP2 correlates well with severity of disease [8,33], provide support for further work aimed at developing measurement of plasma PFHRP2 as an aid to the diagnosis and the management of severely ill African children [25].

Previous reports of field isolates with deletion of one or both of these genes examined samples originating in areas with generally low multiplicity of infection (MOI) [17,18], or focused on samples from individuals with no or few symptoms of malaria and hence likely to have lower MOI [19]. In this survey of around 80 selected parasite samples from patients admitted with a diagnosis of severe malaria but with very low PFHRP2 plasma levels across a number of African countries, there was no evidence for deletion of either *Pfhrp2* or *Pfhrp3*. Multiplicity of infection was not measured, although it is likely that a significant proportion of these samples contained single infections, since around 10% contained insufficient DNA to produce a PCR product in two independent reactions. It is also worth noting that the median asexual parasitaemia in this subgroup was about 9,000 parasites per μl, a figure similar to that associated with false-negative RDTs in the recently published study from Mali. The findings therefore provide a degree of reassurance regarding the prevalence of *Pfhrp2* and *Pfhrp3* deletion across the sites examined.

## Conclusion

PFHRP2 sequence variation in parasites causing severe malaria in Africa does not confound the quantification of PFHRP2 antigen in plasma. This is a key element in the development of plasma PFHRP2 concentration measurement as a method for assessing African children who may have severe falciparum malaria. The evidence supports the concept that sequestered parasite biomass is the major cause of variation of PFHRP2 concentration in patient samples.

## Competing interests

The authors declare that they have no competing interests.

## Authors’ contributions

TR and KP carried out the molecular biology. IH, JM-A, GM, RO, AT, MO, CK, KM, EG, SG, LvS and HR were responsible for clinical data. KS and KC were responsible for parasitological data. TR, IH, CIF, NW, ND, AD, MI and CW participated in the design of the study. TR, KP, MI and CW were responsible for data analysis and management. TR, MI and CW drafted the manuscript. All authors read and approved the final manuscript.
